# An Exploratory Statistical Modeling Framework for National Rule-of-Law Profiles

**DOI:** 10.3390/e28090942

**Published:** 2026-08-22

**Authors:** Sadullah Çelik, Muhammet Ali Köroğlu, Cemile Zehra Köroğlu

**Affiliations:** 1 Department of International Trade and Finance, Aydın Adnan Menderes University, Aydın 09000, Türkiye; sadullah.celik@adu.edu.tr; 2 Department of Social Work, Uşak University, Uşak 64000, Türkiye; cemile.koroglu@usak.edu.tr

**Keywords:** rule of law, institutional performance, statistical modeling, clustering analysis, governance systems

## Abstract

The rule of law can be considered as a multidimensional institutional phenomenon, which emerges through interplay between legal, governance and administrative institutions. The paper offers an exploratory statistical modeling approach to find empirical patterns in national rule-of-law profiles according to the 2024 World Justice Project (WJP) Rule of Law Index. Eight dimensions of the index are considered to identify differences between countries and similarities of their multidimensional institutional performance. Principal Component Analysis reveals strong associations between eight dimensions, which are structured along the same performance institutional scale; the first principal component explains 85.7% of the overall variation and two principal components explain 92.5% of it. K-Means, hierarchical and DBSCAN clustering methods are then used to examine the empirical similarities between countries. While the six-cluster solution of K-Means offers distinct group descriptions, low bootstrap stability of this solution suggests that these groups cannot be regarded as fixed rule-of-law regimes. In addition, the Random Forest analysis reveals Regulatory Enforcement, Absence of Corruption, and Criminal Justice as the three dimensions, which contribute to the empirical differentiation of the described profiles the most. In general, the results imply that international variations in rule-of-law performance are viewed as heterogeneous locations in a multidimensional institution space, rather than as stable and distinct legal systems. The above-presented methodology allows for an exploratory approach to analyze international variations in rule-of-law performance that considers the limitations of cross-section data and instability of clusters.

## 1. Introduction

The rule of law is an important institutional concept which implies that the government has to function in accordance with the law, respect people’s rights, and provide for accountability of its institutions. Not only should there be laws, but also the ability of an institution to enforce the laws, check and balance, transparency, rights protection, and the efficacy of the judiciary. Therefore, the performance of the rule of law across countries can be explained by the differences in other institutional and socioeconomic factors, such as economic development, investments, stability, etc. [1,2,3,4,5].

The problem of understanding why some countries manage to attain better performance levels regarding their institutions, while other countries are characterized by poor performance levels, continues to be relevant in institutional comparisons. The international community, governments, and academics often use rule-of-law indices in comparing institutional performance and making evidence-based evaluations of institutional quality. However, the institutional setting at the country level is multi-dimensional and integrated in nature. It can happen that one country shows better performance in one dimension and much poorer performance in another [6,7,8,9].

The growing availability of international cross-country governance information allows for applying statistical models to analyze complicated institutional phenomena. Since there are several correlated factors that determine the performance in terms of the rule of law, multivariate statistical models can be applied to explore structural connections and empirical patterns that cannot be easily determined when looking at the single factor. Clustering techniques can help to analyze the institutional similarities between different countries with the help of their institutional profile without using any predefined classification.

While quantitative studies on governance and institutional quality have grown substantially, most of the existing literature has concentrated on the study of individual indicators, bivariate correlations, composite indices, or theoretical categorizations of legal regimes. All these have contributed greatly to our knowledge but can potentially fail to account for the multidimensionality of modern rule-of-law practice. Furthermore, in the case where several dimensions of institutions are highly correlated, differences between countries might be due to their different positions in the same institutional performance spectrum rather than to their belonging to different institutional regimes.

From the above standpoint, the current research considers cross-country variability with respect to the eight key components of the 2024 WJP Rule of Law Index. Instead of relying on the assumption of the membership of the examined countries in the different classes of national law and governance, it tries to see whether there are distinct country profiles that can be identified from the multi-dimensional space of rule-of-law performance. The analysis makes use of exploratory data analysis, correlation analysis, principal component analysis (PCA), unsupervised clustering, comparison, and machine learning-based analysis of feature importance.

The study is guided by three interrelated research aims. Firstly, the study will investigate the dimensional architecture of the eight WJP rule-of-law dimensions and whether the variation across countries follows some common institutional architecture. Secondly, the study will investigate whether countries can be empirically profiled according to their similarity in multidimensional rule-of-law performances. Finally, it investigates which dimensions explain the greatest part of empirical clustering of the profiles through additional Random Forest feature importance analysis. Notably, the latter type of analysis is treated more as descriptive than validation since the same set of indicators is used for both clustering and feature importance estimation.

The relevance of this paper can be attributed to its methodological and exploratory contribution to comparative institutional studies. First, the study employs the WJP Rule of Law Index for 2024 to investigate cross-national variations on eight institutional dimensions, rather than just using the overall Rule of Law Score. Second, multivariate dimensional analysis is combined with different unsupervised machine learning techniques to analyze the cross-national similarity structure without applying a priori institutional typology. Third, the empirical profiles obtained in this study are tested for their limitations using internal cluster validation and bootstrapping. Finally, machine learning-based feature importance is used only as a descriptive tool for exploring the dimensions that determine profile differentiation.

The empirical results clearly point out that the eight measures of the rule of law are extremely interrelated and follow a similar scale related to the degree of institution strength and weakness. In this connection, the identified profiles in clustering have to be treated as exploratory empirical groups following the same scale of institution strength/weakness rather than stable and independent regimes of the rule of law in nations. Such a point of view is fundamental for the understanding of the empirical results and considers the cross-sectional nature of the dataset and poor bootstrap stability of the six-cluster solution.

The structure of the paper proceeds as follows. The Section 2 provides the review of the literature on rule-of-law measurement, institutions, and institutional quality, and previous empirical studies of institutional comparison. The Section 3 is devoted to the description of the dataset, variables, and methodology. The Section 4 considers empirical results: exploratory analysis, correlation analysis, PCA, clustering, descriptive analysis, and the additional feature importance analysis. The Section 5 analyzes the results, implications, and limitations. The Section 6 summarizes the principal limitations and further research.

## 2. Literature Review

During recent years, there has been an increasing body of literature concerning the rule of law by researchers from various academic disciplines such as legal studies, politics, governance and development. The existing literature mostly revolves around three major dimensions, which include: the institutional aspects of the rule of law and state capability, as well as the limitation of political power [1,8,9]; the relationship between institutional effectiveness, social trust, and corruption control [3,10]; and the role played by legal institutions in economic and social development [2,11].

While all of this is true, the empirical literature still lacks sufficient research concerning the categorization of countries based on their multidimensional rule-of-law profiles. Indeed, prior research has largely based itself on individual indicators, correlational methods, and institutional dimension reduction by means of factor analysis [12,13,14]. While there have been some attempts to use cluster and network methods for classifying legal systems [15], few researchers have used comprehensive and up-to-date data on the rule of law such as the 2024 World Justice Project (WJP) Rule of Law Index. For this reason, an approach to institutional classification that would be able to reveal latent institutional patterns in different countries is needed [6,7].

The following Table 1 provides an overview of studies that are directly relevant for understanding how the rule-of-law systems have been measured, compared, and quantitatively analyzed in previous research. It is important to mention that none of the studies listed below was chosen because of the results it had produced or because of any theoretical considerations; rather, each of the studies presented in Table 1 has been chosen based on three objective criteria, namely: (i) the usage of internationally comparable rule-of-law or governance indicators, (ii) the contributions made to the empirical measurement or classification of institutional performance, and (iii) the methodological relevancy to the current study.

Based on previous empirical works, it is recommended that the evaluation of the performance of institutions should not just rely on one score since there are various mixes of strengths and weaknesses in an institutional system. Well-performing institutional settings tend to have high state capacity, good public administration, social trust, and democratic accountability [3,11]. On the other hand, poor-performing institutional settings tend to have weak enforcement capacity, corruption vulnerabilities, and frail accountability [31,32].

Nevertheless, the issue of whether different nations manifest multidimensional profiles of their rule-of-law regimes remains empirical. While conventional comparative studies typically depend upon classifying nations according to their broad categories of legal systems, multidimensional statistical methods enable analyzing nations based on similarities and differences in various institutional features [8,24]. Yet, when these features are strongly correlated, their differences might be largely due to different performance levels of countries on a single continuum rather than different institutional regimes. Therefore, an exploratory profiling method offers more insights into cross-country variations without positing any stable institutional categories.

In conclusion, this paper adds to existing research by employing the statistical approach to classification using the latest available 2024 WJP Rule of Law Index data. In contrast to prior approaches that were largely concerned with individual variables, theory-based legal classification, and mere ranking of countries, this research attempts to identify possible commonalities among countries across various institutional dimensions using the data-driven classification approach. Combining the methods of clustering and statistical analysis with machine learning interpretation, this paper provides an empirical approach for studying country-specific rule-of-law performance.

## 3. Methodology and Dataset

### 3.1. Description of the Dataset

The empirical base of this study lies in the WJP 2024 Rule of Law Index. The WJP Rule of Law Index presents a detailed evaluation of the national performance on the rule of law based on the data gathered from population surveys and expert assessments. The index includes eight conceptual dimensions that are evaluated with standardized indicators within a range from 0 (the lowest performance) to 1 (the highest performance) [6].

Cross-sectional design was chosen for this research since the main aim of the study is to determine the current institutional patterns and categorize countries based on their multidimensional rule-of-law indicators rather than analyze change and cause-and-effect dynamics over time. Thus, the 2024 wave of the dataset was chosen as the most current one released by the World Justice Project. A longitudinal analysis of the previous WJP waves will be aimed at addressing another research question.

Below are listed the nine indicators contained in the initial WJP dataset [6,33]:

- Constraints on Government Powers (CGP)
- Absence of Corruption (AC)
- Open Government (OG)
- Fundamental Rights (FR)
- Order and Security (OS)
- Regulatory Enforcement (RE)
- Civil Justice (CJ1)
- Criminal Justice (CJ2)
- Rule of Law Score (WJP)

The composite WJP Rule of Law Index was removed from all clustering analysis and statistical comparisons because it is a composite index which was created using the eight dimensions. Inclusion of the composite index will lead to circularity and increased association among variables. Thus, only the eight dimensions (CGP, AC, OG, FR, OS, RE, CJ1, and CJ2) were used as independent variables for further statistical analysis. The overall WJP index was included in the analysis only for descriptive statistics purposes.

The final sample comprises 140 countries that have complete information on all nine variables. These data were acquired from the WJP database [6,7]. 

### 3.2. Methodological Approach

**Figure 1:**
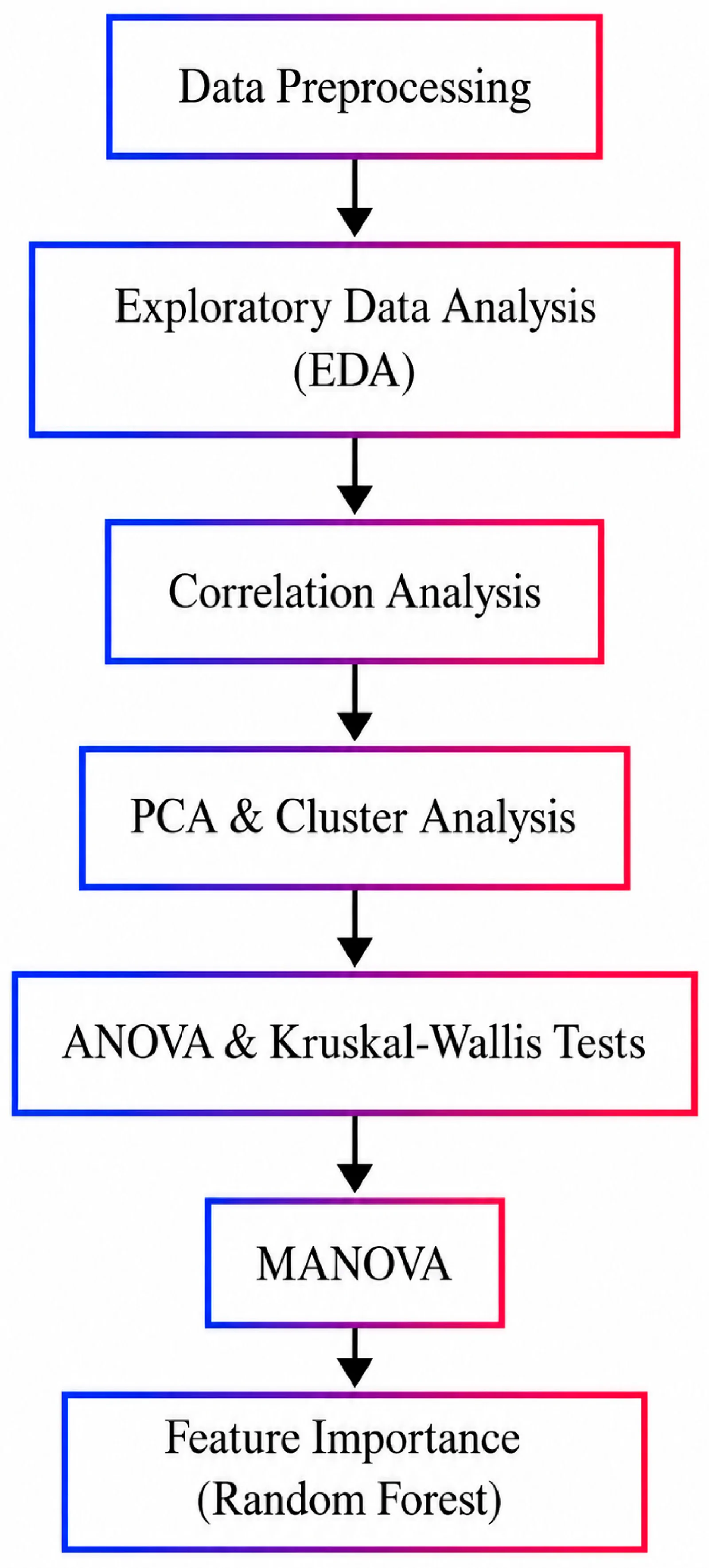
Workflow of statistical analysis methodology.

The objective of this study was to cluster nations into homogeneous groups based on their rule-of-law efficiency and to find out if there were statistically significant differences between these clusters. In order to achieve the goal mentioned above, a multistage methodological approach was used, consisting of data pre-processing, exploratory data analysis, correlation analysis, feature extraction, clustering, parametric and non-parametric tests, MANOVA, and machine learning-based analysis of feature importance. The flowchart depicting the step-by-step stages of the analysis is shown in Figure 1 below.

#### 3.2.1. Step 1: Data Preprocessing

Data preprocessing was done prior to doing cluster analysis based on the WJP Rule of Law Index 2024 dataset to ensure methodological validity and replicability of findings [6]. Specifically, no data normalization was performed since WJP Rule of Law indicators have been already provided in a 0–1 range and do not need any further normalization. All observations with missing values were removed to ensure that only complete cases were used; hence, the final dataset contained data for 140 countries. Data preprocessing included the import of data into Python 3.13 using Pandas 2.3.1, NumPy 2.2.6, and Scikit-learn 1.9.0, followed by validation of the range of indicator values and the presence of missing data. No further identification of countries and their region was preserved for visualization purposes only.

#### 3.2.2. Step 2: Exploratory Data Analysis

EDA has been carried out utilizing the WJP Rule of Law Index 2024 data set to present the differences that may arise from varying rule-of-law performance among countries. Different countries have been ranked based on their total scores. The best and worst countries have been examined through graphical presentation to show their differences. Furthermore, an analysis has been done on each one of the eight factors included in the index by selecting the top- and bottom-ranked countries for each of them individually and representing their performance through a horizontal bar chart.

#### 3.2.3. Step 3: Correlation Analysis

At this juncture, the interrelationships between the main variables forming the WJP were examined with WJP Rule of Law Index 2024 data. The technique of correlation analysis was utilized to determine the strength and direction of the interrelations between the variables, and subsequently, the results were visualized and interpreted. This method was used to identify the extent to which the determinants of the rule-of-law performance are linked and to evaluate the structural interconnections between different indicators.

#### 3.2.4. Step 4: Principal Component Analysis (PCA) and Cluster Analysis

At this stage, PCA was applied to uncover the latent structural dimensions among the eight building blocks of the 2024 WJP Rule of Law Index. PCA is a common dimensionality reduction technique that transforms higher-dimensional data into lower-dimensional data by identifying new orthogonal variables (principal components) that capture the maximum variance in the data set. The largest amount of variance is accounted for by the first principal component (PC1), and subsequent components (e.g., PC2) account for the residual variance orthogonally. The process enables simplification of intricate legal performance data into interpretable dimensions [34,35].

After PCA, three dissimilar unsupervised clustering were utilized with K-Means, Hierarchical Clustering, and DBSCAN to segment countries based on their rule-of-law data. The cluster number was determined through the application of Elbow Method and Silhouette Score, which are classic techniques to test cluster compactness and separability [36].

K-Means is a cluster method that classifies observations into k clusters in such a way that minimizes the sum of the squares of the Euclidean distance between the observations and the corresponding centroid of each cluster. The objective function of the K-Means can be stated as below [37,38,39,40]:

(1) $$\underset{C}{\mathrm{Arg min}}\sum_{i=1}^{k}\sum_{x_{j}\in C_{i}}{\left\|x_{j}-\mu_{i}\right\|}^{2}$$

where $C_{i}$ represents the set of observations assigned to the i−th cluster, $x_{j}\in R^{p}$ denotes the j−th observation represented as a p-dimensional feature vector, $\mu_{i}\in R^{p}$ represents the centroid vector (mean vector) of cluster i, and $∥x_{j}-\mu_{i}{∥}^{2}$ denotes the squared Euclidean distance between the observation vector $x_{j}$ and the corresponding cluster centroid vector $\mu_{i}$ [37,38,39,40].

The algorithm continuously modifies the vectors of centroids and assigns data points to the closest vector of centroids until it converges, meaning that the vectors of centroids do not change any further.

#### 3.2.5. Step 5: Descriptive Comparison of Cluster Profiles Using ANOVA and Kruskal–Wallis Statistics

The generated K-Means clusters were described based on a descriptive comparison of the original variables of the Rule of Law Index. To describe the differences between the generated cluster profiles, one-way ANOVA and the non-parametric Kruskal–Wallis test were calculated separately for each of the eight rule-of-law variables. Prior to calculation, the assumption of normality of distribution was assessed by means of the Shapiro–Wilk test of univariate normality and the Henze–Zirkler test of multivariate normality [41]. Based on the normality diagnostics, it became evident that most variables were not normally distributed. Hence, the Kruskal–Wallis test was presented together with the ANOVA results.

#### 3.2.6. Step 6: Multiple Analysis of Variance (MANOVA)

As the Henze–Zirkler’s test showed that the assumption of multivariate normality of the distribution of the indicators of the rule of law could not be met, the assumptions necessary for conducting MANOVA were not completely fulfilled [41]. For that reason, MANOVA was not used as the key approach for analyzing the differences between clusters. As recommended in the methodology [42], MANOVA was used only as an additional robustness analysis. The core statistical analysis of differences between clusters was made with the Kruskal–Wallis test, suitable for non-normal and heterogeneous data structures [43].

#### 3.2.7. Step 7: Machine Learning-Based Feature Importance Analysis

Random Forest Classifier was applied as an additional supervised learning algorithm to study the impact of eight dimensions of the Rule of Law, CGP, AC, OG, FR, OS, RE, CJ1, and CJ2, in defining the difference between the profiles generated by the K-Means analysis. Random Forest is a machine learning technique, which consists of many decision trees and provides indices of variable importance [40,44]. As clustering and classification have been performed using the same set of variables, it can be said that the obtained results provide the proof of the separation within the clusters rather than an independent or external validation.

## 4. Empirical Results and Findings

### 4.1. Exploratory Data Analysis (EDA)

**Figure 2:**
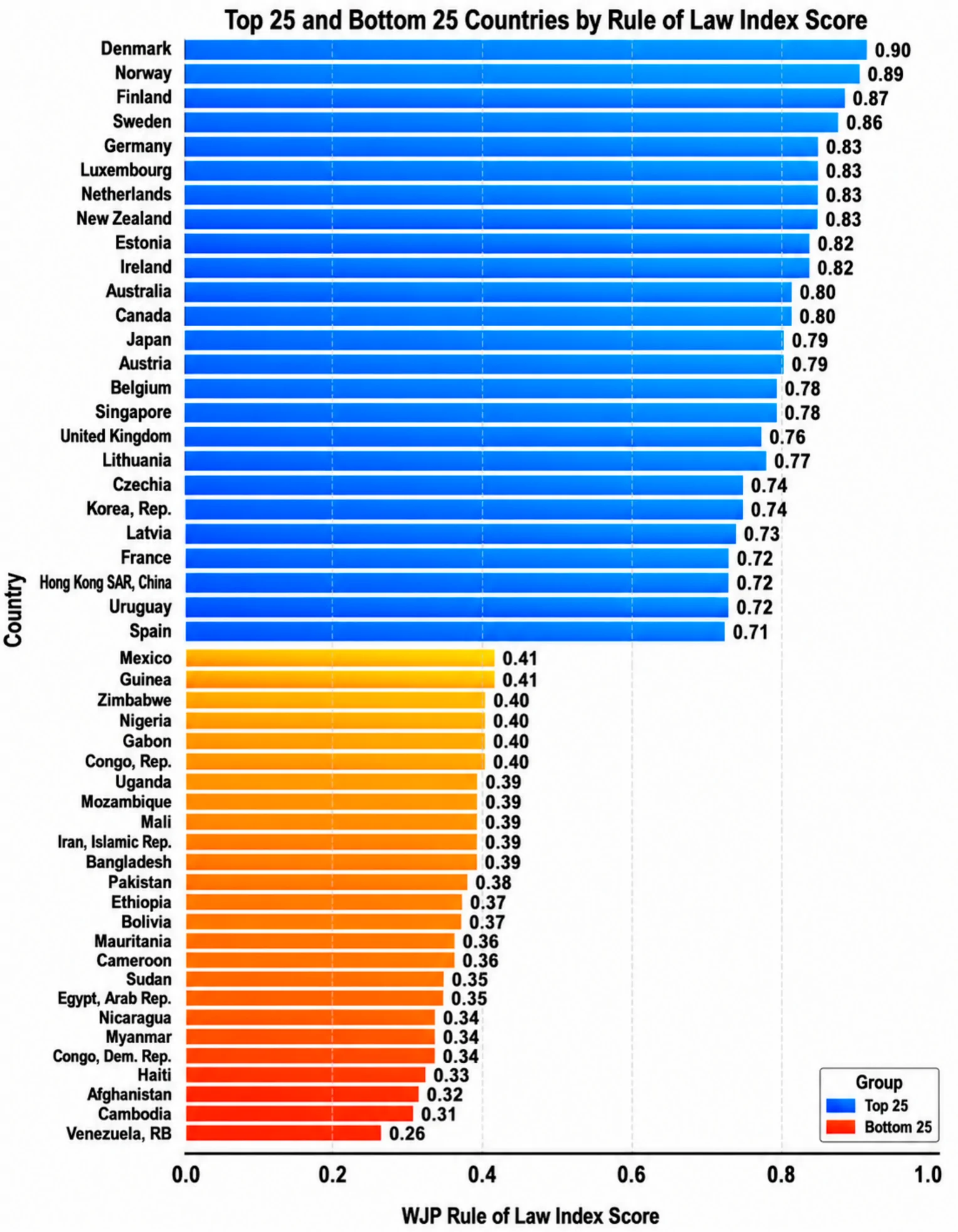
Top 25 and bottom 25 countries by Rule of Law Index score.

In examining the exploratory study of the 2024 WJP Rule of Law Index, there is an evident discrepancy in performance in the extremes of the distribution of the index. As illustrated in Figure 2, the top performers among the 25 countries, particularly those from the Nordic region like Denmark, Norway, and Finland, show scores greater than 0.9 for all the core sub-indicators. It appears that in terms of CGP, AC, and FR, there is a high correlation among the best-performing nations.

**Figure 3:**
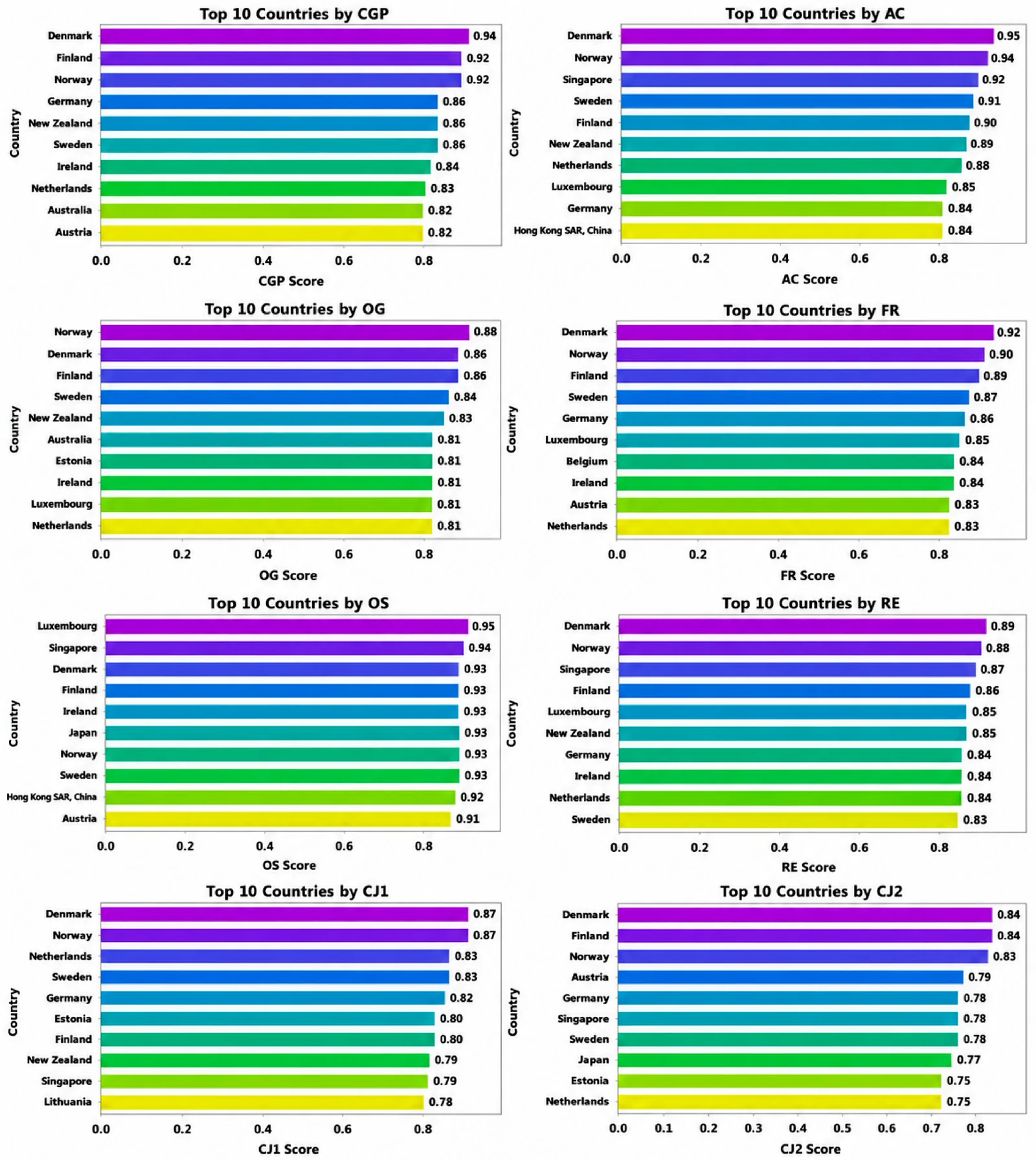
Top 10 countries by rule-of-law factors.

**Figure 4:**
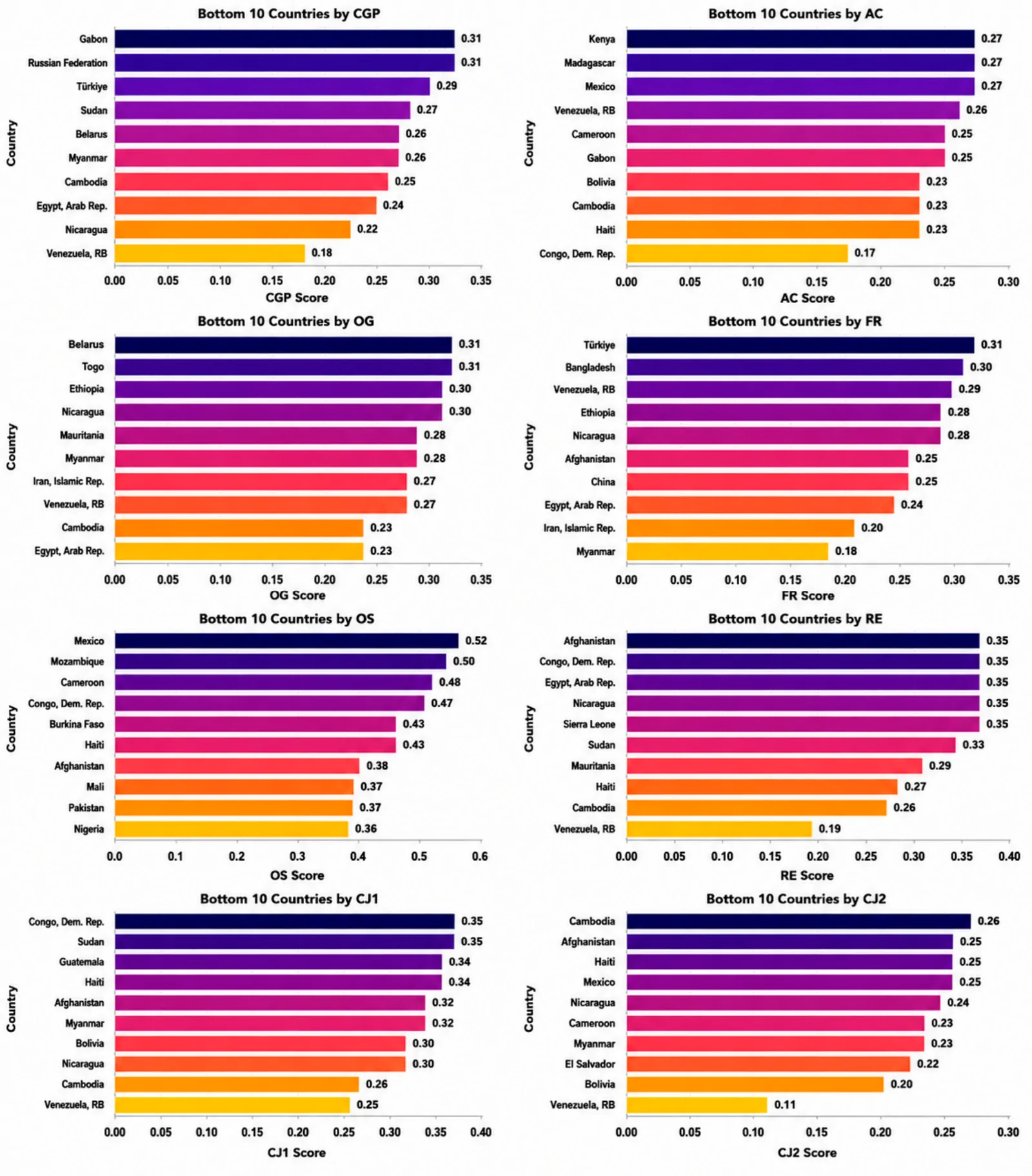
Bottom 10 countries by rule-of-law factors.

On the other hand, the lowest-ranking 25 countries, such as Venezuela, Afghanistan, and Myanmar, have institutional systems that are vulnerable, as evidenced by their factor scores consistently being at or below 0.3. In these countries, the deficiencies are especially pronounced in areas of RE and AC. The consistency of the poor scores in different indicators for these countries, which are represented in Figure 3 below, proves that deficiencies in the rule of law usually do not exist in one particular area but point to more general problems in governance. Figure 3 and Figure 4 highlight the sharp divergence in specific sub-components.

High-Performing Profiles: Both Denmark and Norway top AC and CGP scores at 0.95 and 0.94, respectively, owing to a high level of judicial independence and sound governance controls. The regular occurrence of Nordic countries at the top positions across all eight measures (Figure 3) indicates an overall compliance with rule-of-law principles.

Low-Performing Profiles: From the exploration analysis, it is evident that those countries that have relatively low scores are associated with heterogeneous deficiencies within different rule-of-law dimensions, not a homogenous set of issues. With respect to CGP, the country that obtains the lowest score (0.18) is Venezuela, which is followed by Nicaragua (0.22), Egypt (0.24), and other countries with relatively poor institutional constraint measures. For Türkiye, where the CGP is estimated at 0.29, it is included in the category of lower performers but not the lowest value in this dimension. With respect to institutional integrity and enforcement capacity, countries like the Democratic Republic of Congo, Haiti, Cambodia, and Venezuela have low scores for AC, RE, CJ1, and CJ2.

In Figure 4, the worst-performing countries are presented within the eight dimensions of the rule of law. It is clear from the results that low rule-of-law performance is not associated with the identical institutional setting for all countries; instead, there is a different combination of deficiencies for each country concerning the aspects of government constraint, corruption control, regulation implementation, and judicial performance.

In summary, the results of exploratory data analysis indicate considerable heterogeneity of performance of countries in terms of their adherence to the rule of law. It can be observed that countries with relatively better performance have greater institutional capabilities in different aspects. However, countries with lower performance levels demonstrate different problems in terms of institutional capabilities. The observed diversity serves as an empirical basis for the further clustering analysis.

### 4.2. Correlation Analysis: General Assessment

**Figure 5:**
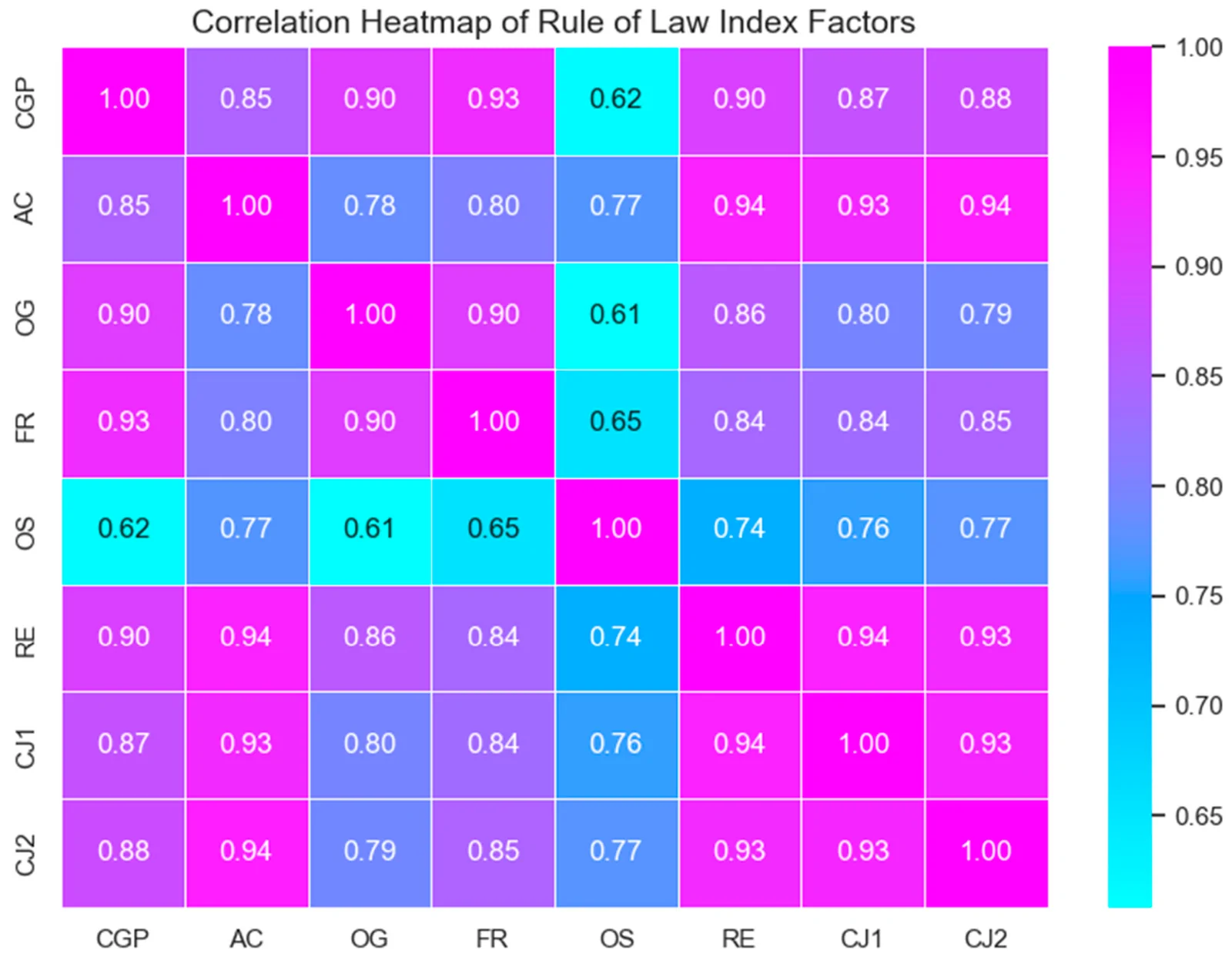
Correlation heatmap of Rule of Law Index factors.

The purpose of carrying out correlation analysis is to determine the structural relations between the eight dimensions of the WJP Rule of Law Index 2024. In order to avoid any possible circularity problem, the composite measure of the WJP Rule of Law Index was excluded from all the analytical methods; the eight dimensions only were used in the correlation analysis. As illustrated in Figure 5, the correlation matrix reveals that there are high positive correlations between most rule-of-law dimensions, showing that the institutional attributes captured by the dimensions are highly interrelated rather than being independent dimensions. The highest correlations are between AC and CJ2 (r = 0.944), AC and RE (r = 0.939), RE and CJ1 (r = 0.939), and CJ1 and CJ2 (r = 0.935).

CGP is also highly correlated with FR (r = 0.926), OG (r = 0.905), and RE (r = 0.898), which reflects the interconnectedness of accountability mechanisms. In turn, OS demonstrates relatively low, though positive, correlations with other dimensions, and the coefficients vary from 0.608 to 0.774. This implies that aspects related to order and security form a relatively separate dimension of the rule-of-law framework. Overall, the findings concerning the correlation of dimensions presented in Figure 5 justify the use of multivariate methods due to the presence of both common structures and specific features of dimensions. Thus, the correlation structure found gives a reason for using PCA and clustering approaches in further stages of the study.

At the same time, lower correlation between OS compared to others in Figure 5 shows that OS can be considered as another aspect of governing that is related to safety but does not necessarily mean institutionalization of transparency and protection of rights. Thus, we may assume that order and security are basic functions of government but are not sufficient for creating an effective rule-of-law environment. Figure 5 supports the concept of mutual influence of institutional dimensions of the rule of law.

### 4.3. Principal Component Analysis

Before clustering the eight rule-of-law dimensions, the PCA was carried out to explore the internal structure and dimensionality of those dimensions. Composite measure of the WJP Rule of Law Index was not included in the PCA procedure as it is an aggregated index based on the very same dimensions and would create redundancy in the analysis. Thus, the PCA was done on the eight standardized dimensions: CGP, AC, OG, FR, OS, RE, CJ1, and CJ2.

**Figure 6:**
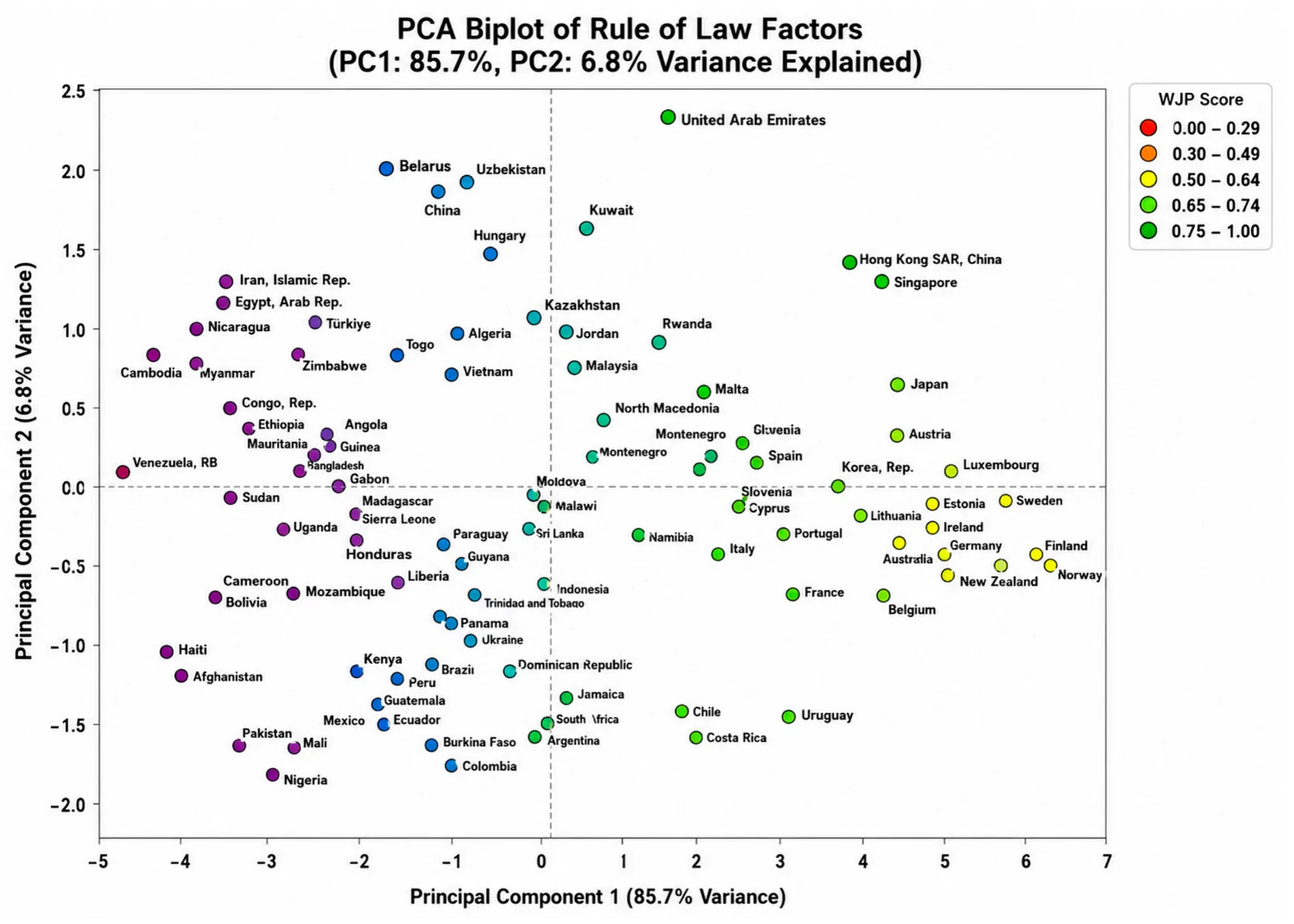
PCA biplot of rule-of-law factors and country groupings based on principal components.

The PCA results indicate a common pattern of structure in the eight dimensions. As illustrated in Figure 6, two first principal components explain 92.5% of the total variability, with 85.7% explained by PC1 and 6.8% by PC2. Such a dominant first component shows that the majority of rule-of-law dimensions mostly represent a common institution strength–weakness dimension rather than separate institutional dimensions. Thus, the PCA suggests that most differences between countries can be explained by their institutional capacity level, while the secondary dimensions’ impact is relatively small.

As is demonstrated in Figure 6, the high PC1 values countries are expected to perform better at several rule-of-law aspects together, namely, institutional accountability, corruption control, judicial effectiveness, and regulatory enforcement. The countries with low PC1 values, on the other hand, would show poor performance in all these interlinked aspects. While there is only one common main axis in PCA structure, the orientation of some vectors may suggest the existence of additional differences between these dimensions. Nevertheless, these differences have to be regarded as variations in an institution’s capacity dimension, not in different governing systems.

While PCA helped to determine the dimensional structure of the problem and visualize it, the following clustering was conducted in an eight-dimensional space of the original variables. This way, descriptive country clusters could be determined without losing any information about the institutional aspect differences.

### 4.4. Cluster Analyses Based on Rule-of-Law Dimensions

The goal of the clustering analysis is to see if there are observable similarities between countries, according to their rule-of-law profiles in eight dimensions. From the PCA results presented in Section 4.3, the dimensions used have a similar institutional capacity framework. This means that the clustering analysis is not expected to uncover different legal regimes, but similarities in the existing institutional space.

**Figure 7:**
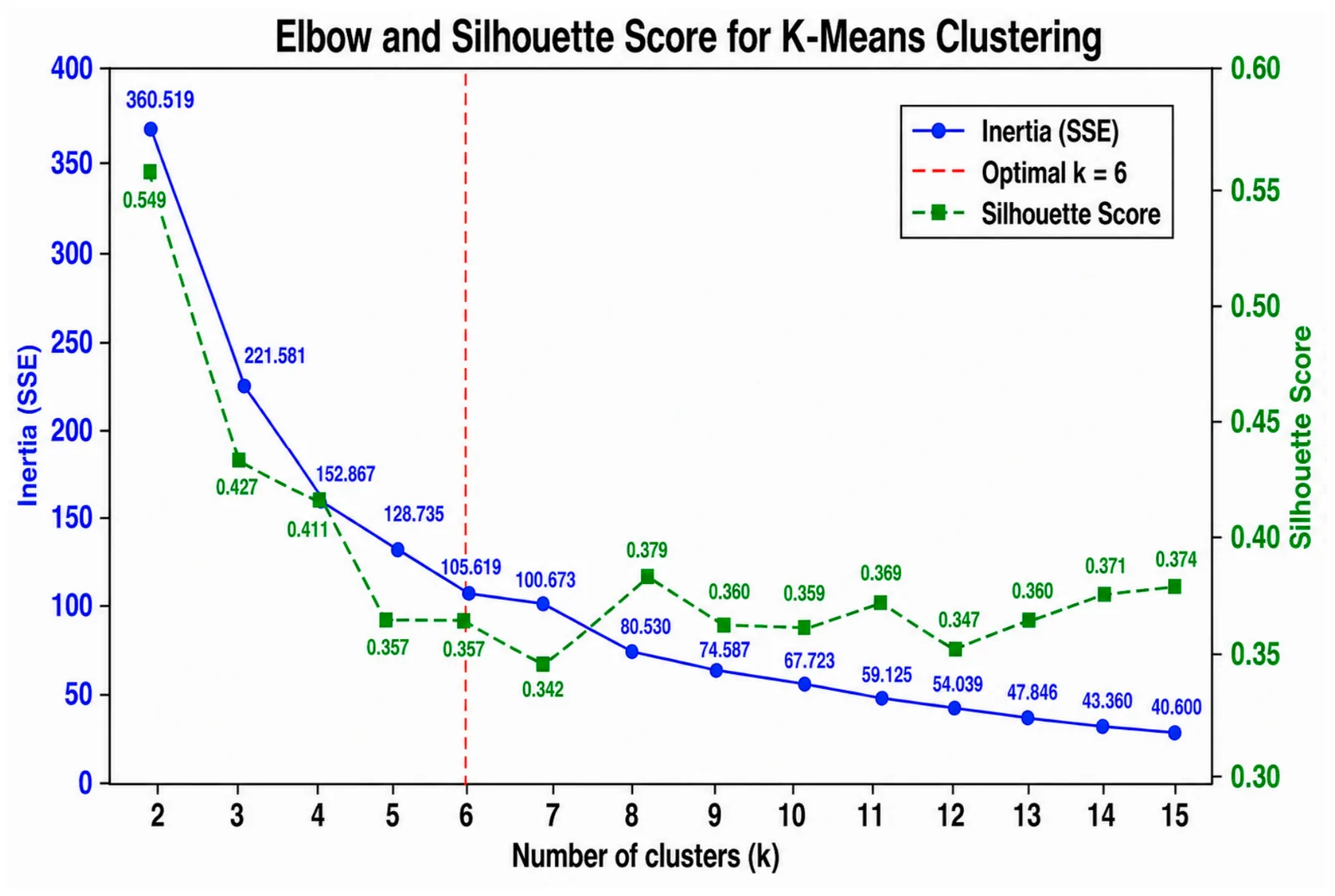
Elbow and Silhouette Score for K-Means clustering.

The number of clusters was investigated using various internal validity measures, such as the Elbow method, Silhouette Score, and Calinski–Harabasz Index (see Table 2 and Figure 7). The highest Silhouette Score was obtained in case of two clusters (k = 2, Silhouette = 0.549), implying that countries can basically be grouped into two different classes in terms of their rule-of-law effectiveness. Nevertheless, since the research seeks to reveal other peculiarities, further cluster options have been considered.

From Table 2, the increase in the number of clusters leads to decreasing intra-cluster variability; however, the separation among the clusters decreases. The solution with six clusters resulted in Silhouette Score equals to 0.357. Even though the maximum Silhouette Score was achieved at (k = 2) (0.549), the solution with six clusters was chosen as an exploratory way of looking at the heterogeneity in the observed space of the performance space of the rule of law. Consequently, the selection of (k = 6) does not mean that there are six different rule-of-law regimes.

**Figure 8:**
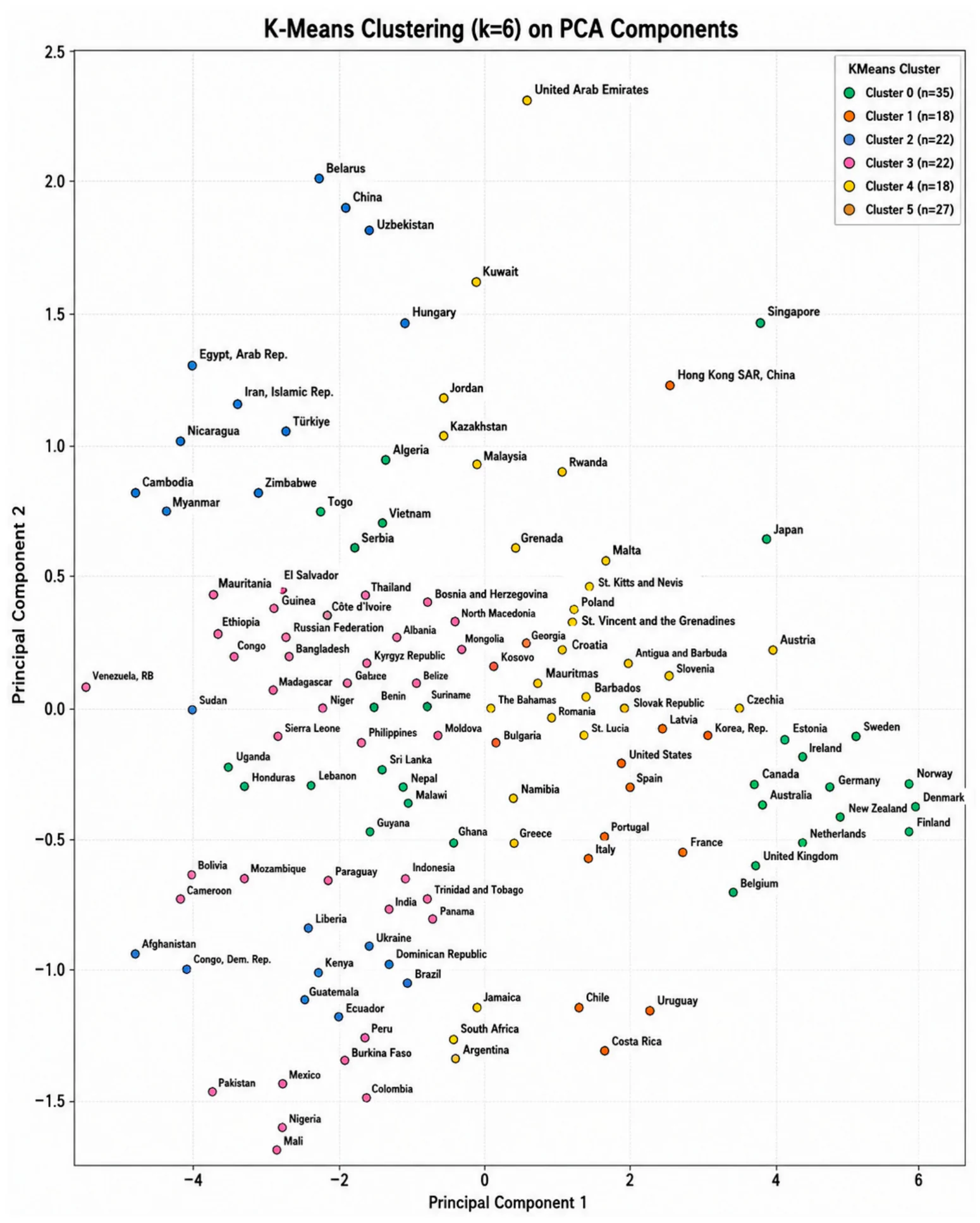
K-Means clustering based on standardized rule-of-law dimensions.

**Figure 9:**
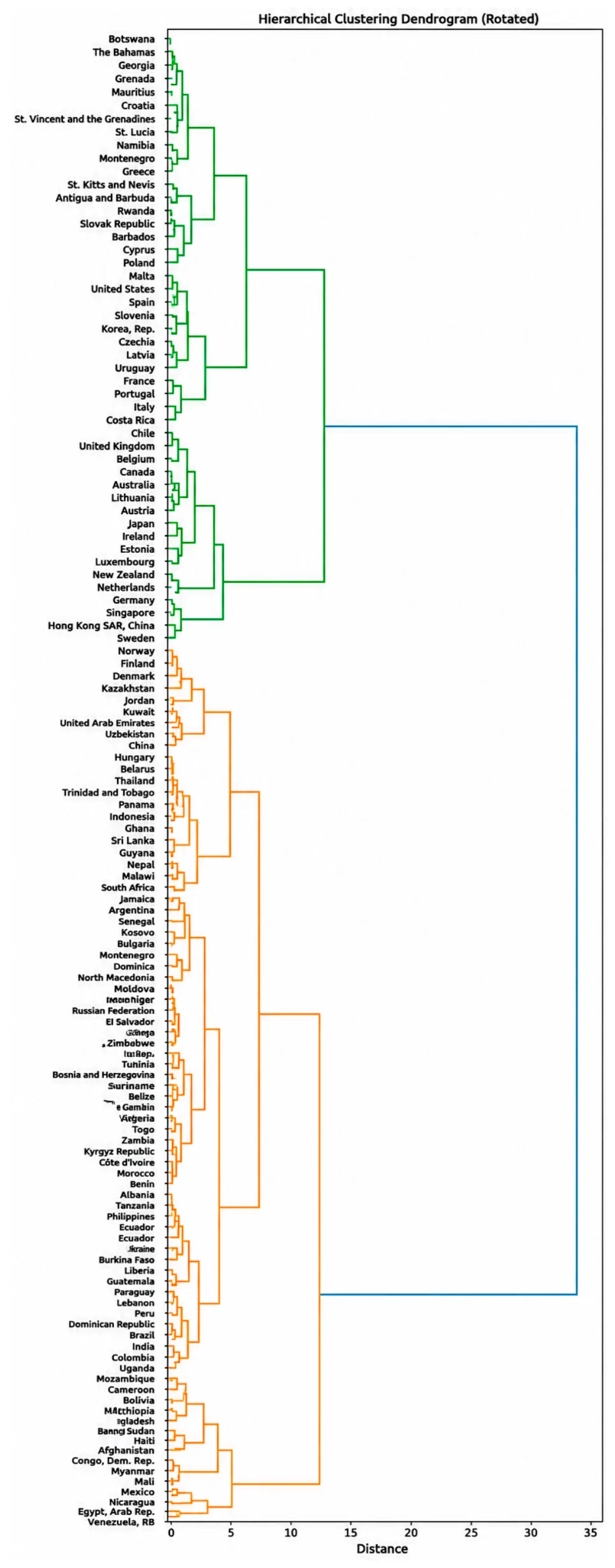
Hierarchical clustering.

**Figure 10:**
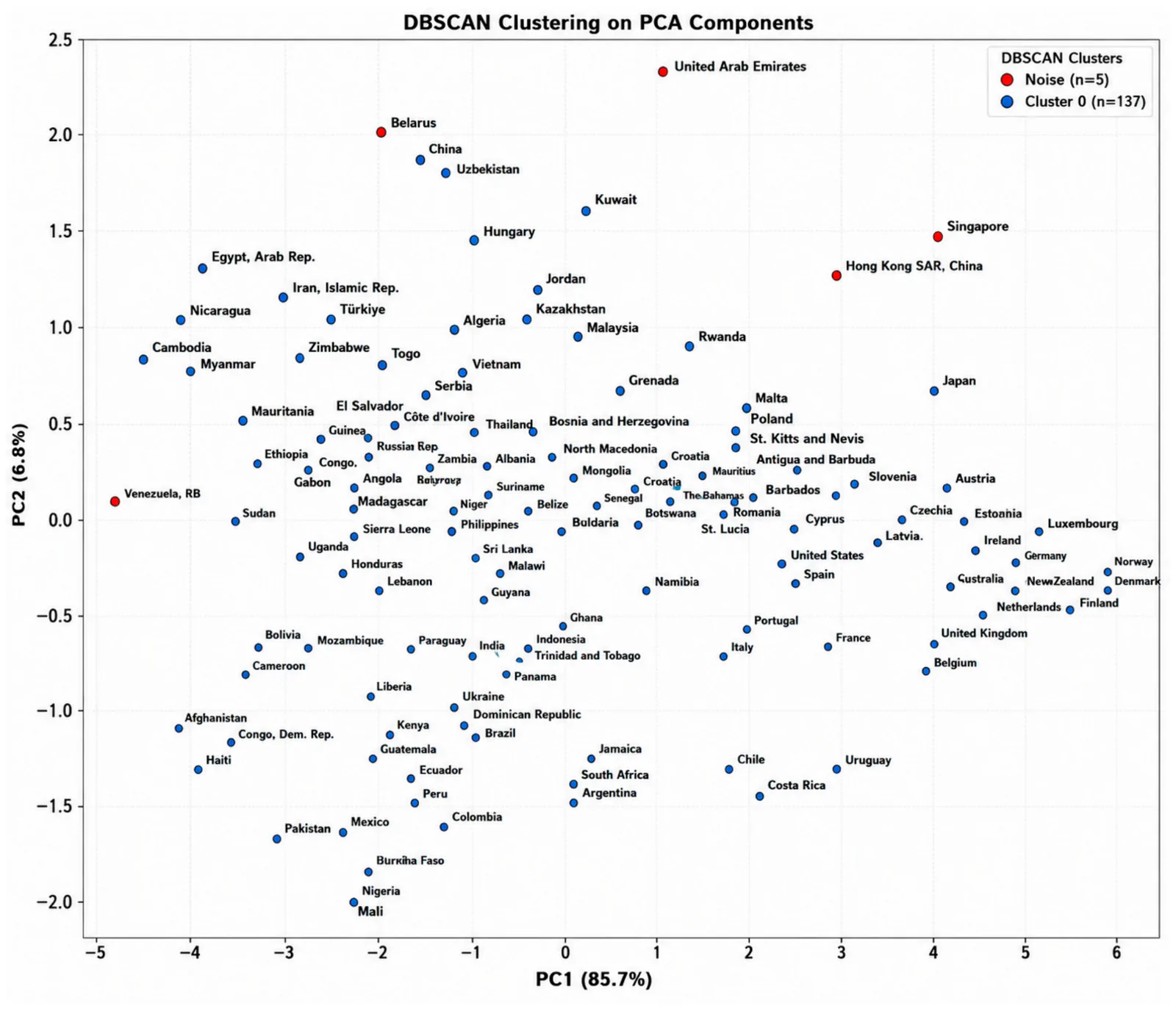
DBSCAN Clustering.

Groupings of countries because of application of the K-Means, Hierarchical Clustering, and DBSCAN algorithms are presented in Figure 8, Figure 9 and Figure 10. While the K-Means clustering algorithm detects several groups of countries with common law profile behavior, the Hierarchical Clustering algorithm yields different groupings which, however, have some similarities to the previous groupings. DBSCAN, on the other hand, detects only one large cluster of observations along with several isolated observations. This can be seen as an empirical grouping in the observed rule-of-law performance space.

The assessment of the performance of clustering algorithms was based on the calculation of the values for internal validity indices as well as bootstrap stability (Table 3). In terms of internal validation criteria, K-Means yielded significantly higher Silhouette and Calinski–Harabasz indices compared to other clustering methods. Nonetheless, these indices cannot guarantee the stability of the resulting clusters.

Nonetheless, the bootstrap stability findings show serious drawbacks of the six-cluster solution. Both the ARI score (−0.001) and Jaccard score (0.091) imply very poor stability under sampling. In that case, the six-cluster solution cannot be understood as the final classification of the rule-of-law systems of nations. On the contrary, this solution can only be treated as an exploratory set of empirical profiles that reflect similarities between nations on the scale of the 2024 WJP Rule of Law Index.

In general, the cluster analysis shows significant variation in rule-of-law performance profile clusters between different countries. But due to the low bootstrap stability of the six-cluster solution, such profiles have to be treated carefully as exploratory empirical clusters and not as stable institutional clusters. Thus, further analysis is based on description of the differences between the empirical clusters revealed by the observed data. For the validation of these results, the longitudinal data of the WJP and other institutional indicators would be needed.

**Figure 11:**
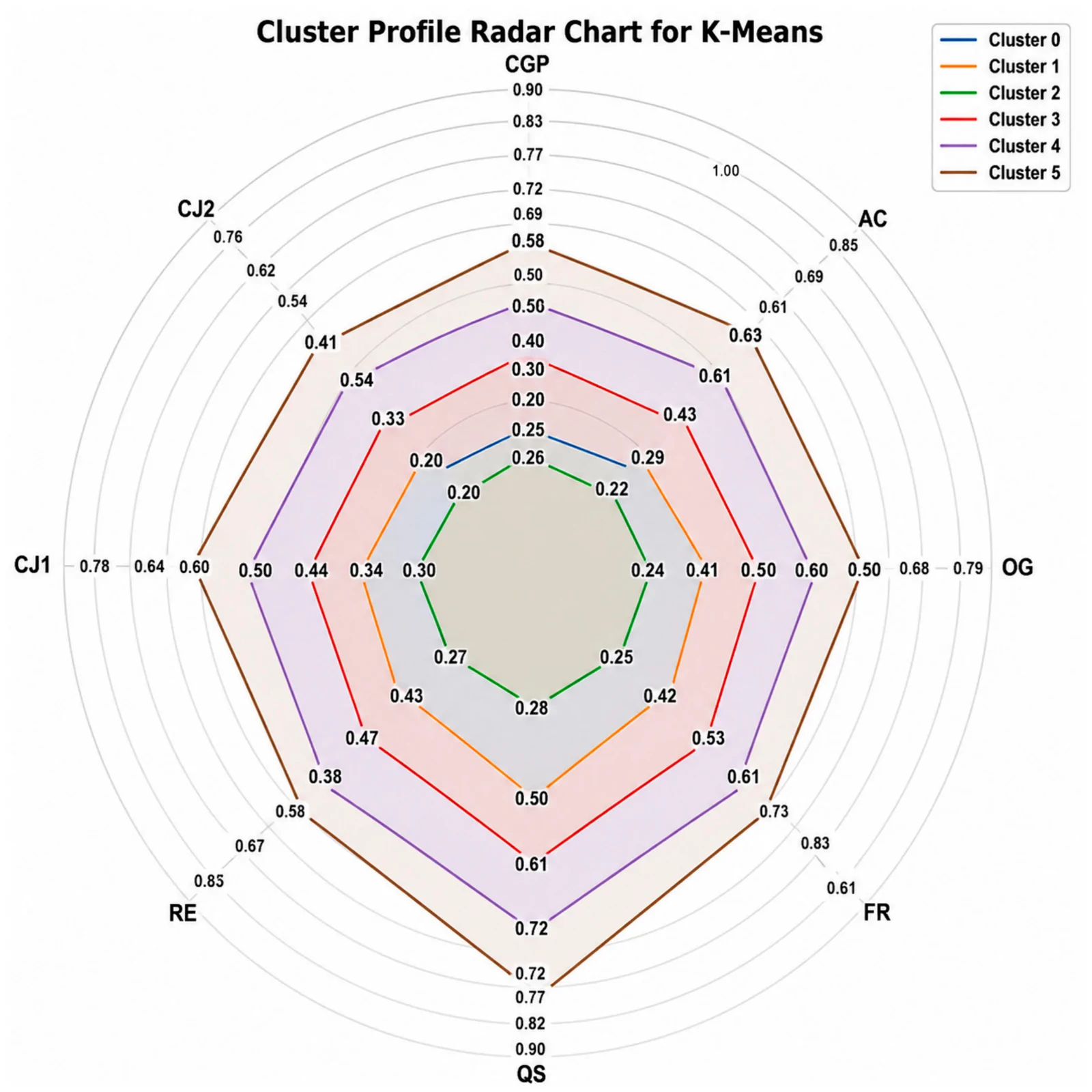
Cluster Profile Radar Chart for K-Means.

Figure 11 shows the average profiles of the six K-Means clusters. As one can see from Figure 11, there is an important difference between the countries belonging to various clusters when it comes to certain rule-of-law dimensions such as regulatory enforcement, judicial performance, and civil rights.

The patterns seen in Figure 11 reinforce the clusters seen in Figure 8 and provide additional information about the attributes that separate the clusters into groups. Together, Figure 8, Figure 9, Figure 10 and Figure 11 and Table 2 and Table 3 indicate that countries have significant differences in the performance of the rule of law. Nevertheless, considering the relatively poor cluster quality indicators and bootstrap stability indicators, along with the cross-sectional nature of the data, it is advisable to be cautious when drawing policy conclusions from the results obtained.

### 4.5. Descriptive Comparison of Cluster Profiles

Having determined the six K-Means clusters, descriptive statistical comparisons were performed to illustrate the differences between the original rule-of-law dimensions about their cluster profile. The idea behind these statistical tests was to describe the institutional profile represented by each of the clusters rather than provide an independent validation of the clustering algorithm employed. Therefore, the statistical data reported below must be considered as descriptive rather than as independent validation of the clustering procedure applied.

Before proceeding to these descriptive comparisons, the distributional assumptions of the rule-of-law variables were tested using the Shapiro–Wilk test for normality. As shown in Table 4, only the variables CGP (*p* = 0.0612) and FR (*p* = 0.0953) could be considered as approximately normally distributed, while the others showed statistically significant departures from normality (*p* < 0.05). It suggests that most of the rule-of-law dimensions are not normally distributed. Thus, apart from the traditional parametric tests, non-parametric statistical measures are justified. In addition, this observation correlates with the violation of the multivariate normality assumption discovered in the methodology evaluation.

Table 4 contains the results of the Shapiro–Wilk normality test for the eight dimensions of the rule of law. The results show that, for most of the variables, the assumption of normal distribution is not fulfilled, except for the CGP and FR variables, where the null hypothesis of normality cannot be rejected. Considering that most of the variables have non-normal distributions, it is more appropriate to use the Kruskal–Wallis test to compare the differences between clusters. The results of one-way ANOVA are also provided for descriptive purposes but not considered as primary evidence for the analysis. Furthermore, since the clusters were constructed based on the same rule-of-law dimensions analyzed here, the differences found are an indication of the internal separation between the clusters, not of their validity as such.

It is evident from the results presented in Table 5 that all the rule-of-law dimensions have different statistically distinguishable distributions for the discovered cluster profiles (all *p* < 0.001). Nevertheless, the significance values mentioned just reflect the differences between the clusters in terms of the initial institutional variables and are not the proof of statistical validation of the algorithm used to identify them. Thus, Table 5 reflects merely the composition of the institutions included in each cluster profile.

**Figure 12:**
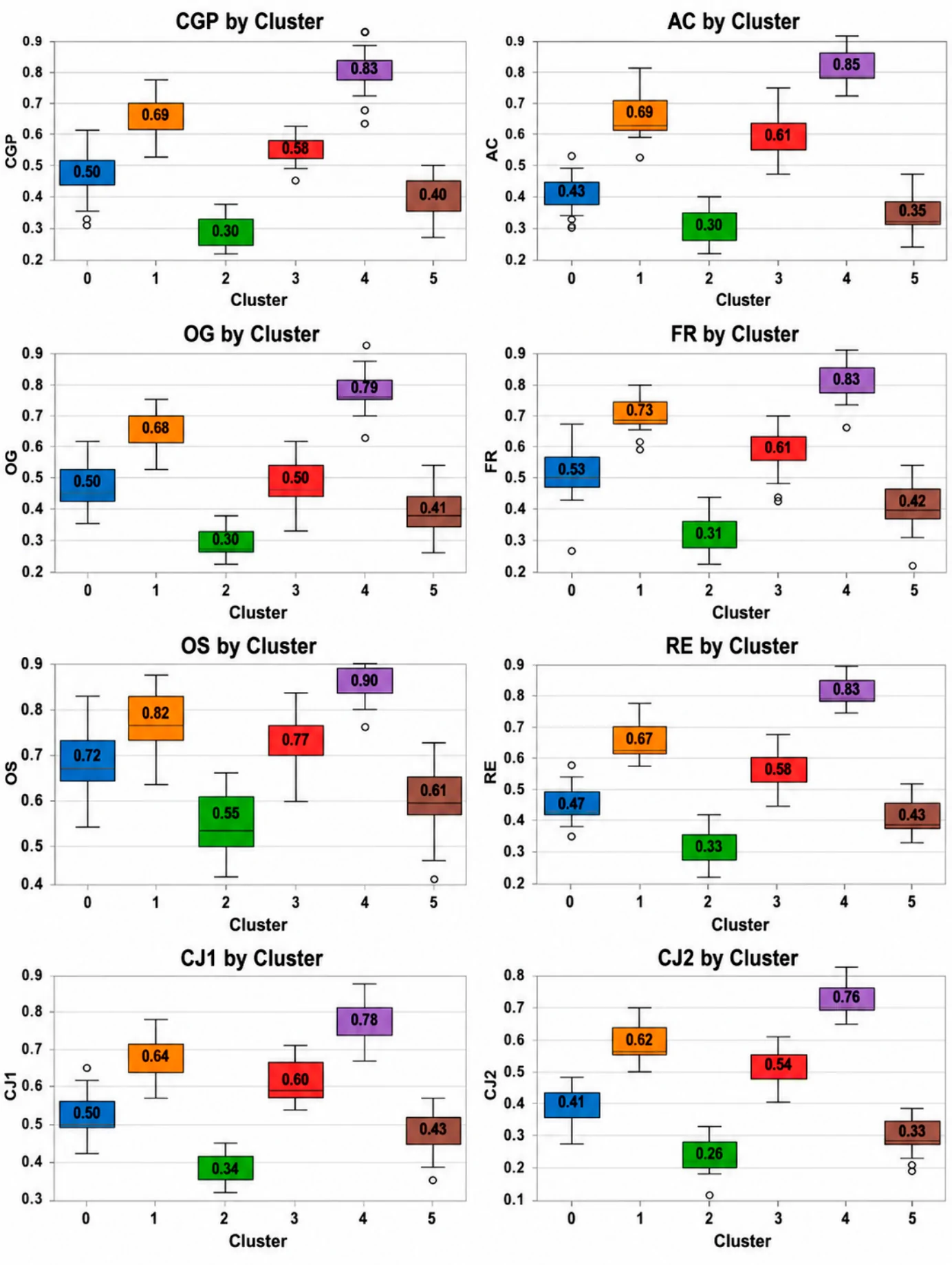
Boxplots of legal indicators by K-Means cluster with mean values annotated.

The attributes of the empirical profile that have been observed are given in Figure 12, wherein the box plots have the distribution of all the eight dimensions of the Rule of Law Index in the six K-Means clusters. As shown in Figure 12, there are variations in the medians, inter-quartile range, and the variation for some of the dimensions such as Regulatory Enforcement, Absence of Corruption, and Criminal Justice.

In conclusion, the results presented in Table 4 and Table 5, along with graphical descriptions provided in Figure 12, give an overall picture of the characteristics of institutions that are related to the clusters. These results represent differences in the clusters described based on the available data set but cannot be used for the validation of the K-Means clustering solution.

### 4.6. Descriptive Multivariate Comparison of Cluster Profiles Using MANOVA

In order to add to the description of the distributions of each of the rule-of-law dimensions through univariate descriptive statistics described above, a multivariate analysis of variance (MANOVA) was performed. The goal of the latter was not inferential but rather descriptive and thus should be understood in the context of the exploratory nature of this study.

Prior to the interpretation of the MANOVA output, the assumptions behind the procedure were examined. It was established that one of those assumptions, the assumption of multivariate normality, was violated, as indicated by the Henze–Zirkler (1990) [41] multivariate normality test. Therefore, MANOVA cannot be viewed as an inferential procedure in the process of cluster validation. Furthermore, since the variables used in constructing the K-Means clusters are the same as those included in estimating the MANOVA model, it is expected by default to observe statistically significant multivariate differences.

As shown in Table 6 below, the descriptive multivariate statistics suggest that the cluster profiles are distinct in the joint distribution of the rule-of-law components (Wilks’ Λ = 0.8151, F = 3.3274, *p* = 0.0011). It is important to note that these descriptive statistics only describe the characteristics of the clusters identified using multivariate analysis and do not serve as any form of inference regarding the clustering procedure used.

In conclusion, the findings obtained from Table 6, combined with those from the univariate analysis conducted in Table 4 and Table 5 and visual analysis in Figure 12, confirm that the profiles found empirically differ in the joint distribution of the dimensions of the rule of law. Yet, these findings are not sufficient to validate the solution through independent analyses. Hence, all cluster memberships throughout this thesis will be treated in an exploratory and descriptive way due to the observed differences between institutional performances.

### 4.7. Machine Learning-Based Feature Importance Analysis

To describe the dimensions that are responsible for the empirical separation of the K-Means profiles, an additional Random Forest classifier was used. It is worth noting that the goal of such descriptive analysis was not to confirm the quality of the clustering results or to predict the results with high accuracy, but to analyze the relative influence of the eight rule-of-law dimensions on the separation of the empirically obtained country profiles. It is important to distinguish between these two types of analysis since the same eight indicators were used for constructing the K-Means clusters and the Random Forest classifier.

The Random Forest model was built using the eight dimensions that were included in the K-Means analysis: CGP, AC, OG, FR, OS, RE, CJ1, and CJ2. Given that the cluster labels were produced using these eight dimensions, the feature-importance values should be regarded as descriptive characteristics of the cluster solution and not as its causal explanation.

**Figure 13:**
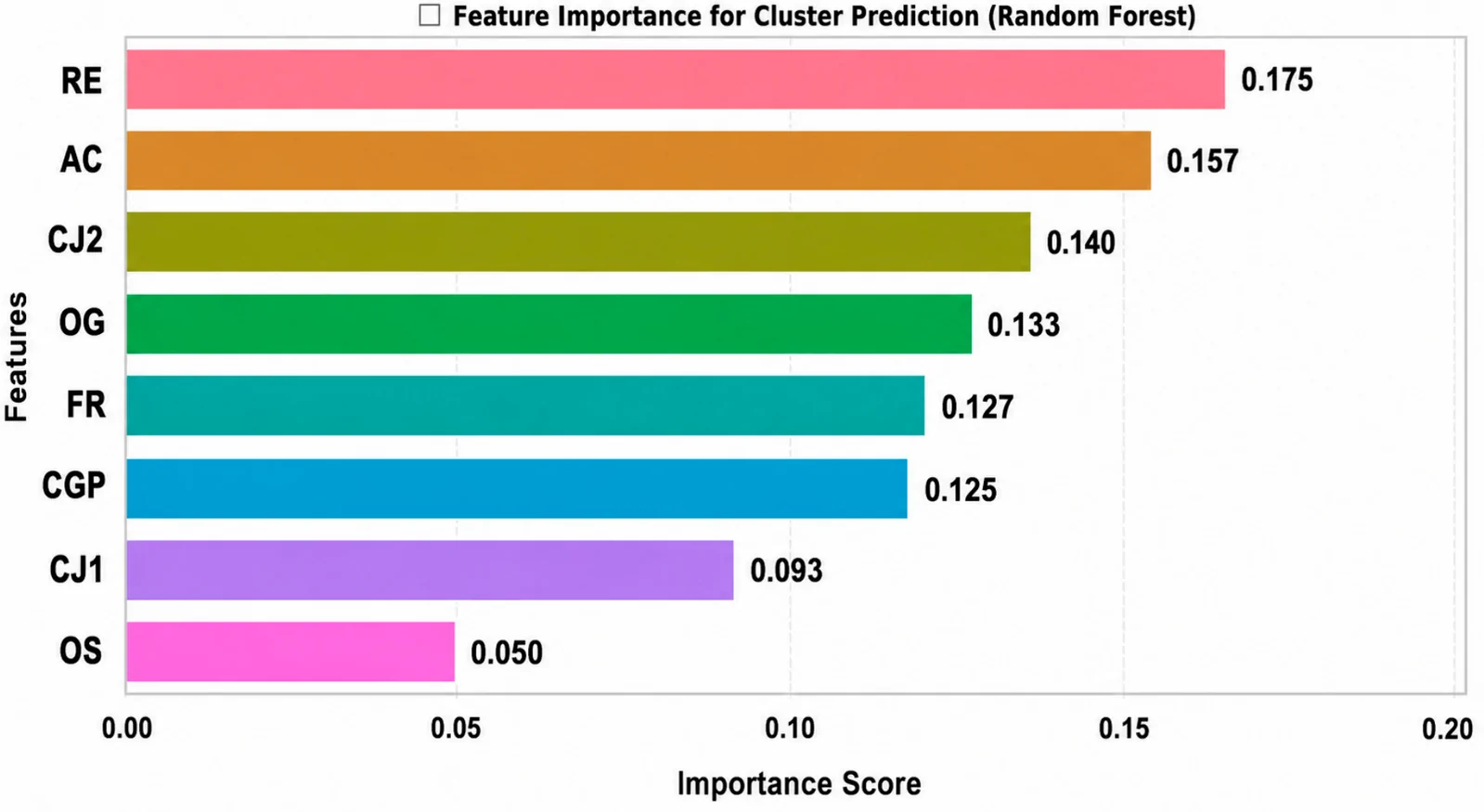
Relative feature importance of rule-of-law dimensions in Random Forest-based cluster profiling.

Based on the feature-importance results illustrated in Figure 13, it is possible to note that RE, AC, and CJ2 are the three most important dimensions that contribute the most to the separation of the K-Means profiles. The total contribution to the separation of K-Means profiles made by the three most important dimensions is 47.2%. This means that the discrepancies in terms of enforcement capacity, institutional integrity, and criminal justice performance are especially evident in the multidimensional rule-of-law space of the clusters created. Nevertheless, the importance of these dimensions is associated with the correlation within the created cluster space only.

**Figure 14:**
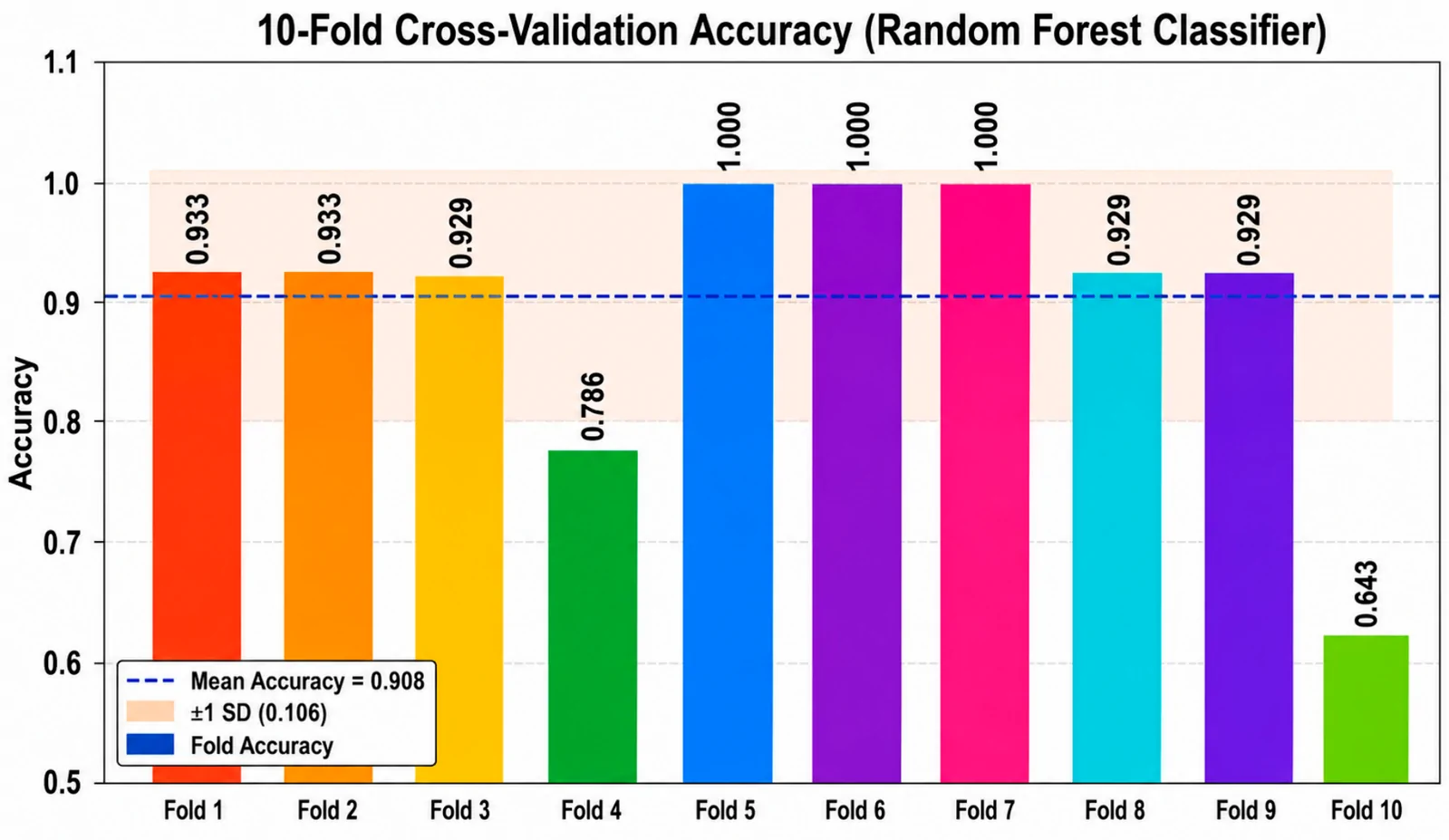
Ten-fold cross-validation accuracy for the descriptive assessment of internal cluster separability.

In addition to the empirical information regarding the internal separability of the profiles, a tenfold cross-validation analysis was also performed. The fold-specific classification accuracies were in the range 0.643 to 1.000 with the mean accuracy of 0.908 and the standard deviation of 0.106 (Figure 14). Only the mean cross-validation accuracy is presented in this paper as an additional measure of the consistency of cluster labels obtained from the same eight rule-of-law dimensions. This figure should not be treated as external validation or prediction, nor as a sign of stability of the six-cluster profile solution.

It is crucial for the current research that the distinction be made between the feature importance as a descriptive measure and the cross-validation accuracy as the measure of independence of the results. Since both K-Means clustering and Random Forest classification are based on the same eight institutional dimensions, the Random Forest is learning the separation that is already present in the feature space used for the generation of cluster labels.

Combining these findings gives descriptive information regarding the nature of the observed empirical profiles. As RE, AC, and CJ2 are the three most significant factors, these findings suggest that the ability to enforce laws, control corruption, and provide criminal justice are especially relevant for the differentiation of the countries in the rule-of-law performance space. Nevertheless, it should be mentioned that these findings do not prove causality, external validity, or distinct governance regimes.

Consequently, the Random Forest approach employs yet another exploratory instrument for investigating empirical profiles instead of verifying a preconceived classification or testing the hypotheses about different governance regimes. As can be seen from the results, which are in accordance with correlation and PCA, there is a high degree of association between the eight rule-of-law dimensions, which are mainly arranged according to the institutional performance axis. Therefore, the discrepancies between country profiles can be regarded as empirical discrepancies along the institutional performance axis rather than stable governance regimes.

The use of longitudinal World Justice Project data, other measures of institutions, and independent measures of outcomes in future research will allow a more robust assessment of whether such profiles are stable over time, as well as whether the importance of the dimensions identified is stable across institutions.

## 5. Conclusions and Discussion

The present research aimed to analyze cross-country differences in the rule of law in terms of the eight dimensions in the 2024 WJP Rule of Law Index. To do so, K-Means cluster analysis was used to empirically differentiate the countries based on their similarities and differences in institutional performance across the multidimensional space. It is apparent that there exists a great diversity in rule-of-law performance among countries; however, the PCA shows that all dimensions are mostly aligned in terms of one axis of institutional performance.

Although there are statistical significant differences between the profiles empirically observed in each of the analyzed dimensions according to the ANOVA and Kruskal–Wallis tests, the extremely low bootstrap ARI and Jaccard statistics show the instability of the six-cluster solution. Therefore, although the clusters give descriptive insight into the differences in the rule-of-law performance space, the allocations of specific countries can depend on sample variation. The six clusters should be considered an empirical and exploratory grouping of countries’ rule-of-law regimes.

In order to analyze further the dimensions involved in the empirical distinction of the country profiles, the Random Forest feature importance analysis was conducted. Since the classification was conducted based on the same eight dimensions of the Rule of Law Index used for clustering in the K-Means analysis, the results of the analysis were not taken as indicative of any predictive or independent validation of the empirically observed profiles. Rather, they are seen as descriptive analysis pointing to the dimensions which are the most important in their distinction. The results show that RE, AC, and CJ2 are the three most important dimensions involved in the differentiation of the empirical rule-of-law profiles.

With regard to policy implications, the clusters found offer an empirical basis through which it is possible to understand varying institutional profiles and areas that may need some attention. Nonetheless, it is important to keep in mind that any policy implications drawn will have to take into consideration the constraints posed by the cross-sectional and single-source nature of the data used. The countries that belong to the better-performing clusters could be concerned with sustaining their institutional quality, while also working on areas where further improvements can still be made, especially in the aspect of enforcement and judicial effectiveness.

The discovered clusters also indicate that there are different constitutional configurations in various countries. High-capacity clusters exhibit strong institutional coordination and accountability processes, while low-capacity clusters indicate weak institutional processes on various grounds. However, such interpretation of the findings needs to be regarded as descriptive in nature, which does not provide any explanation for the development paths of nations. Thus, the findings also contribute to the wider understanding that the performance of rules of law is a multidimensional concept based on the interplay of institutional factors.

Consequently, the present work has contributed to the methodology literature on comparative institutional studies by employing unsupervised learning techniques, inferential statistics, and explainable machine learning approaches to discover heterogeneous rule-of-law profiles of different countries. The suggested approach represents a reproducible method for analyzing the institutional system, taking into consideration the problem of cluster instability and cross-sectional data structure. In the future, it is recommended to explore how stable these institutional structures are over time using longitudinal databases.

## 6. Limitations

There are several factors to consider while evaluating the results of this research. First, the analysis is limited by the 2024 WJP Rule of Law Index. Hence, it is only a cross-sectional study that examines the patterns of rules of law in various countries. Even though data collection allows for constructing a thorough and internationally consistent set of measurements, a one-year observation is not sufficient for tracking institutional changes or reform dynamics and assessing the stability of the rule-of-law pattern.

The second point is that the obtained clusters need to be perceived as exploratory institutional profiles of the countries’ rule-of-law systems, not as any conclusive or fixed classifications of such regimes. Despite the better clustering efficiency for the K-Means clustering method, the bootstrap stability analysis shows that the six-cluster solution is unstable under variations in data.

Lastly, the results of the clusters depend on the choice of indicators, methodology, and features of the dataset used. Therefore, the obtained clusters need to be regarded as the analytical classification of similar countries based on the similarities and differences found. Future studies may continue developing this approach by including longitudinal data and additional indicators for clustering.

## Figures and Tables

**Table 1:** Overview of previous empirical studies on rule-of-law measurement and comparative analysis.

Authors	Insights	Methodology	Findings
Foweraker and Krznaric (2003) [16]	This paper examines the differentiation of democratic performance among Western democracies.	Descriptive statistics to compare democratic performance measures.	Western democratic performance is neither uniform nor superior. Significant variation in rights.
Voigt (2012) [8]	Evaluation of the rule of law based on eight distinct dimensions.	Bivariate correlations across eight rule-of-law indicators.	Low correlations suggest diverse strengths across countries.
Møller and Skaaning (2013) [13]	Empirical links between rule-of-law sub-components and reduced subtypes.	Principal component analysis of sub-indicator relationships.	High inter-correlations; diminished subtypes lack empirical validity.
Siems (2016) [15]	Classifies global legal systems into clusters using network analysis.	Network analysis applied to 156-country legal system data.	Identifies four major clusters; supports legal transplant and harmonization analysis.
Andrrs Jakab and Lorincz (2017) [17]	Methodological critique of rule-of-law index construction.	Emphasizes transparent index design and data aggregation strategies.	Importance of reducing subjectivity in EU rule-of-law metrics.
Versteeg and Ginsburg (2017) [18]	Comparison of four leading rule-of-law indicators.	Correlation analysis and judicial independence benchmarking.	High indicator correlation; convergence due to shared measurement strategy.
Teodoro Lara (2017) [19]	Explores development–rule of law relationship in Brazil, Italy, U.S.	Comparative analysis of governance and development indicators.	Rule of law positively correlates with GDP and sustainable development.
Andras Jakab and Lorincz (2020) [20]	Applies rule-of-law indices to EU rule-of-law crisis.	Use of international indices and statistical monitoring tools.	Methodology crucial for EU legal accountability frameworks.
Shevchuk, Blikhar, Komarnytska, & Tataryn (2021) [21]	Examines rule-of-law and GDP growth links in 41 countries.	World Justice Project index and regression models.	Rule of law enhances GDP growth in certain post-Soviet states; inflation dampens growth.
Berendieieva et al. (2022) [22]	Analyzes public interest, governance, and rule of law.	Content analysis and hermeneutic methods.	Rule of law supports public interest; index decline does not devalue concept.
Pai et al. (2023) [23]	Rule of law’s effects on migration, unemployment, and crime.	Panel data regression across multiple nations.	Strong inverse link with crime index; unemployment and migration increase crime.
Horák and Lacko (2023) [24]	Triangulates theoretical and empirical rule-of-law concepts.	Mixed-method triangulation using legal conceptualizations and indicators.	Reveals disconnect between theory and measurement practices.
Kabat-Rudnicka (2023) [25]	Studies rule of law across national and supranational levels.	Comparative legal analysis.	EU governance best fits English rule-of-law model.
Brad (2024) [26]	Investigates economic effects of rule-of-law deterioration.	Empirical comparative analysis (USA, Singapore, Romania).	Rule-of-law decline weakens economic growth; corruption suppresses prosperity.
Closa and Hernández (2025) [27]	Evaluates EU rule-of-law enforcement preferences among states.	Qualitative Comparative Analysis (QCA).	Enforcement linked to democracy quality and EU budget stance.
Jelić and Jungfleisch (2025) [28]	Links between rule of law and anti-corruption mechanisms.	Legal-empirical analysis of ECtHR jurisprudence.	Rule of law undermined by corruption; recovery policy suggested.
Sun (2025) [29]	Examines rule of law in environmental governance.	Spatial governance models + rule-of-law framework.	Rule of law ensures effectiveness of ecological protection regimes.
Kalaitzaki (2025) [30]	Assesses parliaments’ role in EU value promotion.	Institutional analysis of Cypriot/EU democratic processes.	Democratic standards in parliaments critical for rule-of-law adherence.

**Table 2:** Inertia (SSE) and Silhouette Scores for different cluster sizes.

K	Inertia (SSE)	Silhouette Score
2	360.519	0.549
3	221.581	0.427
4	152.867	0.411
5	128.735	0.357
6	105.619	0.357
7	100.673	0.342
8	80.530	0.379
9	74.587	0.360
10	67.723	0.359
11	59.125	0.369
12	54.039	0.347
13	47.846	0.360
14	43.360	0.371
15	40.600	0.374

**Table 3:** Clustering methods performance comparison.

Method	Silhouette Score	Calinski–Harabasz Index	Davies–Bouldin Index	Bootstrap ARI	Bootstrap Jaccard
K-Means	0.357	243.452	0.879	−0.001	0.091
Hierarchical	0.331	208.521	0.836	–	–
DBSCAN	0.244	1.442	3.686	–	–

**Table 4:** Shapiro–Wilk normality test results for the eight rule-of-law dimensions.

Feature	Test Statistic	*p*-Value	Normality Conclusion
CGP	0.9822	6.12 × 10^−2^	Normally distributed (fail to reject H0)
AC	0.9455	2.36 × 10^−5^	Not normally distributed (reject H0)
OG	0.9656	1.23 × 10^−3^	Not normally distributed (reject H0)
FR	0.9839	9.53 × 10^−2^	Normally distributed (fail to reject H0)
OS	0.9702	3.42 × 10^−3^	Not normally distributed (reject H0)
RE	0.9364	4.99 × 10^−6^	Not normally distributed (reject H0)
CJ1	0.9765	1.51 × 10^−2^	Not normally distributed (reject H0)
CJ2	0.9603	3.98 × 10^−4^	Not normally distributed (reject H0)

**Table 5:** ANOVA and Kruskal–Wallis test results for the eight rule-of-law dimensions across clusters.

Feature	ANOVA *p*-Value	Kruskal–Wallis *p*-Value
CGP	1.6563 × 10^−54^	1.1076 × 10^−23^
AC	1.3630 × 10^−66^	1.9909 × 10^−24^
OG	2.8433 × 10^−47^	4.1664 × 10^−21^
FR	1.1347 × 10^−48^	5.5576 × 10^−23^
OS	1.0382 × 10^−27^	1.3476 × 10^−18^
RE	6.2258 × 10^−70^	9.2612 × 10^−25^
CJ1	1.5994 × 10^−58^	2.7697 × 10^−24^
CJ2	1.7709 × 10^−65^	5.8835 × 10^−25^

**Table 6:** MANOVA results for cluster differences based on rule-of-law indicators.

Test	Value	Num DF	Den DF	F Value	Pr > F
**Intercept**					
Wilks’ lambda	0.0418	9	132	336.0423	0.0000
Pillai’s trace	0.9582	9	132	336.0423	0.0000
Hotelling–Lawley trace	22.9120	9	132	336.0423	0.0000
Roy’s greatest root	22.9120	9	132	336.0423	0.0000
**Cluster**					
Wilks’ lambda	0.8151	9	132	3.3274	0.0011
Pillai’s trace	0.1849	9	132	3.3274	0.0011
Hotelling–Lawley trace	0.2269	9	132	3.3274	0.0011
Roy’s greatest root	0.2269	9	132	3.3274	0.0011

## Data Availability

The dataset and Python codes used in the analysis from the link below: https://github.com/Sadullah4535/Rule-of-Law-Index. (accessed on 12 July 2026).
